# Association of immune‐related pneumonitis with clinical benefit of anti‐programmed cell death‐1 monotherapy in advanced non‐small cell lung cancer

**DOI:** 10.1002/cam4.4045

**Published:** 2021-06-13

**Authors:** Kana Ono, Hirotaka Ono, Yukihiro Toi, Jun Sugisaka, Mari Aso, Ryohei Saito, Sachiko Kawana, Tomoiki Aiba, Tetsuo Odaka, Suguru Matsuda, Shin Saito, Akane Narumi, Takahiro Ogasawara, Hisashi Shimizu, Yutaka Domeki, Keisuke Terayama, Yosuke Kawashima, Atsushi Nakamura, Shinsuke Yamanda, Yuichiro Kimura, Yoshihiro Honda, Shunichi Sugawara

**Affiliations:** ^1^ Department of Pulmonary Medicine Sendai Kousei Hospital Sendai Miyagi Japan

**Keywords:** anti‐programmed cell death‐1, checkpoint inhibitor pneumonitis, immune‐related adverse events, non‐small cell lung cancer, outcome

## Abstract

**Background:**

The association between the development of checkpoint inhibitor pneumonitis (CIP) with tumor response and survival has remained unclear so far. The aim of the present study was to evaluate the association between CIP and the clinical efficacy of anti‐programmed cell death‐1 antibody in patients with advanced non‐small cell lung cancer (NSCLC).

**Methods:**

Between January 2016 and August 2019, 203 advanced NSCLC patients were administered with nivolumab or pembrolizumab. Comparisons were made between patients with and without CIP. We evaluated the time‐to‐treatment failure (TTF), progression‐free survival (PFS), and overall survival (OS).

**Results:**

CIP was observed in 28 (14%) patients. CIP was associated with a longer PFS (18.9 months [95% confidence interval, CI: 8.7 months–not reached] vs. 3.9 months [95% CI: 3.4–5.1 months, *p* < 0.01]) and longer OS (27.4 [95% CI: 20.7 months–not reached] vs. 14.8 months [95% CI: 11.2–17.9 months, *p* = 0.003]). Most patients discontinued the immune checkpoint inhibitor (ICI) treatment when they developed CIP. Seven patients (25%) lived for more than 300 days from treatment discontinuation and did not show any long‐term tumor growth after treatment discontinuation.

**Conclusion:**

CIP was associated with prolonged PFS and OS. Additionally, 25% of CIP patients did not show any tumor growth for long periods after treatment discontinuation. Careful management of CIP can help in obtaining the best clinical efficacy from anti‐PD‐1 antibody.

## Graphical abstract

This study aimed to evaluate the association between checkpoint inhibitor pneumonitis (CIP) and the clinical efficacy of anti‐programmed cell death‐1 monotherapy in patients with advanced non‐small cell lung cancer (NSCLC). CIP was associated with a longer PFS (18.9 months vs. 3.9 months, *p* < 0.01) and longer OS (27.4 vs. 14.8 months, *p* = 0.003), and 25% of CIP patients did not grow tumors long after treatment was discontinued. We believe that cautious management of irAEs (especially early detection and treatment) can facilitate achieving the maximum clinical benefit from nivolumab or pembrolizumab monotherapy.

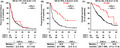

**Lay summary**

Checkpoint inhibitor pneumonitis (CIP) is one of the immune‐related adverse events (irAEs). To date, an association between CIP and better tumor response or clinical outcome remains unclear. In our study, CIP was associated with progression‐free survival (PFS) and overall survival (OS). Also, 25% of CIP patients did not have tumor progression long after treatment discontinuation. Most importantly, appropriate management was performed at the time of CIP onset. We think that careful management of CIP might maximize the clinical benefit of nivolumab and pembrolizumab monotherapy.

## 1 INTRODUCTION

Lung cancer is the most common type of cancer‐related death worldwide.^1^ Recently, programmed cell death 1 (PD‐1) and programmed cell death ligand 1 (PD‐L1) inhibitors (alone or in combination) have been shown to result in higher survival rates than standard chemotherapy in patients with advanced NSCLC.^2^, ^3^, ^4^, ^5^, ^6^, ^7^, ^8^ Administration of immune‐checkpoint inhibitors (ICIs) is complicated by immune‐related adverse events (irAEs) including pneumonitis, skin reactions, thyroid dysfunction, hepatitis, and infusion reaction; these differ from the adverse events of conventional systemic therapy.^9^ Some studies have reported that the occurrence of irAEs is linked to the clinical efficacy of ICIs in patients with NSCLC.^10^, ^11^, ^12^ However, it is unknown which irAE is particularly linked to the clinical benefit of ICIs.

Among individuals with irAEs, 5%–10% of the patients treated with ICIs developed checkpoint inhibitor pneumonitis (CIP), resulting in potentially serious toxicity.^13^ Similar to other irAEs, low‐grade CIP was found in most cases, and it improved with immunosuppressive therapy. However, severe CIP can lead to fatal respiratory failure.^14^ The onset of CIP may be a reflection of the degree of immune activity, but it is currently unclear whether CIP development is an indicator of better antitumor response or clinical efficacy. We performed a retrospective study to investigate the association between CIP and the clinical efficacy of nivolumab or pembrolizumab in advanced NSCLC patients.

## 2 MATERIALS AND METHODS

### 2.1 Patients

We performed a single‐institutional retrospective study of the medical records of patients with advanced NSCLC treated with either nivolumab or pembrolizumab between January 2016 and August 2019 at Sendai Kousei Hospital.

### 2.2 Assessment

We analyzed CIP, skin reaction, infusion reaction, thyroid dysfunction, and hepatitis as irAEs. We defined irAEs as adverse events that require more frequent monitoring and may have an immunological basis requiring intervention with immunosuppression and/or endocrine replacement therapy.^15^ Patients were assigned to two groups (with or without CIP), and we evaluated their time‐to‐treatment failure (TTF), progression‐free survival (PFS), and overall survival (OS). The best tumor response was defined with reference to the Response Evaluation Criteria in Solid Tumors (Ver. 1.1).^16^ We evaluated the clinical severity of irAE according to the Common Terminology Criteria for Adverse Events, version 4.0.

### 2.3 Statistical analysis

Categorical variables were evaluated utilizing chi‐square, Student's *t*‐test, Mann–Whitney *U* tests, or Welch's *t*‐test where appropriate. Survival outcome was estimated with the Kaplan–Meier curves and was compared between patient groups with the log‐rank test. The relationship between patient variables and response was evaluated with univariate and multivariate logistic regression analyses. The Hazard ratios (HRs) were estimated using Cox proportional hazards. All *p* values were two‐sided, and those <0.05 were considered statistically significant.

All statistical analyses were performed with EZR (Saitama Medical Center, Jichi Medical University, Saitama, Japan).^17^

This study was approved by the Sendai Kousei Hospital Institutional Review Board (IRB No. 30–36). Data were analyzed anonymously; therefore, there was no need to obtain informed consent from patients.

## 3 RESULTS

### 3.1 Patient characteristics

Two hundred and three patients (146 men [72%]; 57 women [28%]) received nivolumab (n = 141) or pembrolizumab monotherapy (n = 62) (Table 1). The median age was 70 (range 31–92) years. One hundred and ninety‐five (96%) patients had an Eastern Cooperative Oncology Group Performance Status (PS) of 0 or 1. Eighty‐one (40%) and 122 (60%) patients were diagnosed with squamous cell carcinoma and non‐squamous NSCLC, respectively. Epidermal growth factor receptor mutations were expressed in 21 (10%) patients. Thirty‐eight (19%) patients had not been on any prior chemotherapy regimen, whereas 91 (45%), 37 (18%), and 37 (18%) patients had received 1, 2, or ≥3 courses of chemotherapy. PD‐L1 was expressed in abundance (tumor proportion score ≥50%) in 51 (25%) patients, expressed at low levels (1% ≤ tumor proportion score <50%), absent (tumor proportion score <1%), and unknown in 152 (75%) patients. No patient had any active autoimmune disease and interstitial lung disease.

**TABLE 1:** Patient characteristics at baseline (n = 203)

Characteristics	Value^a^
Age, years	70 [31–92]
Sex (male), No. (%)	146 (72%)
ECOG PS	
0	120 (59%)
1	75 (37%)
2	8 (4%)
Smoking	
Current or past smoker	168 (83%)
Never smoked	35 (17%)
Pathological subtype	
Squamous cell carcinoma	81 (40%)
Non‐squamous NSCLC	122 (60%)
Mutated EGFR	21 (10%)
Nivolumab/pembrolizumab monotherapy, No.	141/62
Prior chemotherapy regimens	
0	38 (19%)
1	91 (45%)
2	37 (18%)
≥3	37 (18%)
History of thoracic radiotherapy	38 (19%)
PD‐L1 expression	
TPS ≥50% (strong positive)	51 (25%)
1% ≤ TPS <50% (weak positive)	48 (24%)
< TPS 1% (negative)	38(19%)
TPS unknown	66(32%)
Development of irAEs, No. (%)	110 (54%)
Development of severe irAEs, No. (%) (Grade≧3)	17 (8%)
Onset of irAEs, weeks	8.7 [1–80.0]

Abbreviations: ECOG PS, Eastern Cooperative Oncology Group performance status; irAEs, immune‐related adverse events; NSCLC, non‐small cell lung cancer; EGFR, epidermal growth factor receptor; TPS, tumor proportion score.

^a^ Median [range] or number (%).

^b^ Scores range from 0 to 4, with high numbers indicating high disability.

Complete response was seen in 3 (1%) patients, partial response in 57 (28%) patients, stable disease in 76 (38%) patients, and progressive disease in 67 (33%) patients. The objective response rate (ORR) was 30% (95% confidence interval [CI] 23–36), whereas the disease control rate was 67% (95% CI 60–73).

Immune‐related adverse events.

The irAEs are summarized in Table 2. Of the 110 patients with irAEs, 58 (29%) patients presented with skin reactions, whereas 29 (14%), 28 (14%), 19 (9%), and 12 (6%) patients developed thyroid dysfunction, CIP, infusion reaction, and hepatitis, respectively.

**TABLE 2:** Immune‐Related adverse events in the study (n = 110)

Variables	n	Onset time (week), Median	CTCAE ver. 4.0 Grade, n 1/2/≧3	Tumor Response, n(%) CR/PR/SD/PD	Objective response rate (%)
Skin reaction	58(29%)	6.4	41/14/3	2/29/23/4	53%
Pneumonitis	28(14%)	20	5/16/7	0/19/8/1	68%
Infusion reaction	19(9%)	0	13/6/0	1/8/6/4	47%
Thyroid dysfunction	29(14%)	7.8	16/11/2	0/13/10/6	45%
Hepatotoxicity	12(6%)	8	8/3/1	0/5/6/1	42%

Abbreviations: CTCAE, Common Terminology Criteria for Adverse Events; CR, complete response; PR, partial response; SD, stable disease; PD, progression disease.

The ORR was significantly better in patients who developed CIP and skin reactions than in those without CIP (68% vs. 24%, *p* < 0.001 and 53% vs. 20%, *p* < 0.001, respectively) (Table 2).

Table 3 shows the predictors of ORR, PFS, and OS in the multivariate analysis. CIP was also an independent predictor of ORR, PFS, and OS (ORR odds ratio 9.45 [95% CI: 3.35–26.6, *p* < 0.01]; PFS, HR 0.31 [95% CI: 0.18–0.56, *p* < 0.01]; OS, HR 0.31 [95% CI: 0.16–0.60, *p* < 0.01]).

**TABLE 3:** Multivariate analysis of ORR, PFS, and OS

	ORR			PFS			OS		
	Odds ratio	95% CI	P^a^	HR	95% CI	P^b^	HR	95% CI	P^b^
Sex (male)	0.86	0.33–2.26	.77	1.08	0.72–1.63	.70	1.28	0.78–2.08	.33
Age	1.00	0.96–1.05	.97	1.00	0.98–1.02	.90	1.01	0.99–1.03	.29
ECOG PS	0.75	0.38–1.46	.40	1.18	0.88–1.57	.26	1.81	1.32–2.50	<.001
Smoking (current/past)	2.2	0.59–8.25	.24	0.87	0.54–1.41	.57	1.10	0.62–1.95	.75
Prior chemotherapy regimens	0.71	0.47–1.07	.10	1.06	0.95–1.18	.31	1.16	1.03–1.29	.01
Mutated EGFR, positive	2.15	0.35–1.32	.41	1.86	1.09–3.16	.02	0.74	0.38–1.46	.39
Nivolumab/pembrolizumab	0.99	0.41–2.43	.99	1.02	0.70–1.48	.93	1.19	0.77–1.84	.44
Skin reaction	5.13	2.37–11.1	<.001	0.40	0.27–0.58	<.001	0.40	0.25–0.63	<.001
Infusion reaction	2.05	0.64–6.60	.23	0.86	0.50–1.50	.60	0.53	0.26–1.08	.08
Pneumonitis	9.45	3.35–26.6	<.001	0.31	0.18–0.56	<.001	0.31	0.16–0.60	<.001
Thyroid dysfunction	2.47	0.88–6.92	.09	0.69	0.43–1.03	.12	0.54	0.29–0.97	.04
Hepatitis	0.75	0.18–3.12	.69	1.09	0.58–2.05	.78	0.95	0.43–2.07	.89

Abbreviations: irAE, immune‐related adverse event; ORR, objective response rate; PFS, progression‐free survival; OS, overall survival; PS, performance status; EGFR, epidermal growth factor receptor.

^a^ Results calculated with logistic regression.

^b^ Results calculated with Cox proportional hazard model.

**FIGURE 1:**
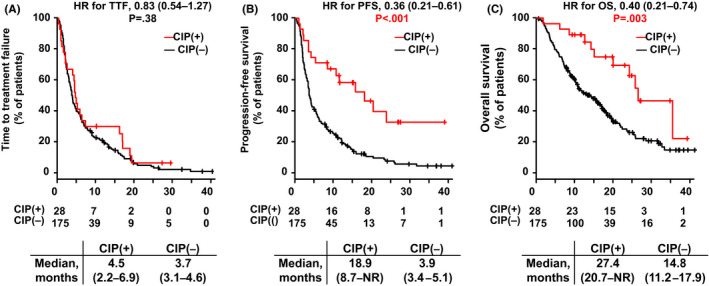
Time‐to‐treatment failure, progression‐free survival, and overall survival in the study population. Kaplan–Meier curves are shown for time‐to‐treatment failure (A), progression‐free survival (B), and overall survival (C) in patients with or without checkpoint inhibitor pneumonitis (CIP). The red line; with CIP; the black line; without CIP

Figure 1 shows a comparison of TTF, PFS, and OS for patients with and without CIP. CIP was associated with longer PFS (18.9 months [95% CI: 8.7–not reached] vs. 3.9 months [95% CI: 3.4–5.1 months, *p* < 0.01]). In addition, CIP was associated with longer OS (27.4 [95% CI: 20.7 months–not reached] vs. 14.8 months [95% CI: 11.2–17.9 months, *p* = 0.003]).

**FIGURE 2:**
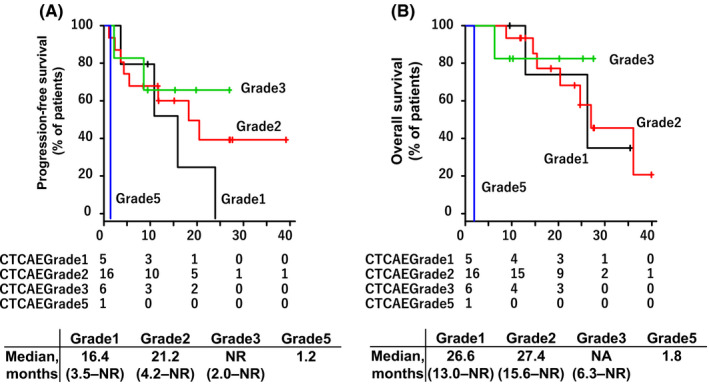
PFS and OS in patients with checkpoint inhibitor pneumonitis by Grade. Kaplan–Meier curves are shown for progression‐free survival (A) and overall survival (B) in patients with checkpoint inhibitor pneumonitis by Grade. The black line; Grade 1; the red line; Grade 2; the green line; Grade 3; the blue line; Grade 5

Figure 2 shows the PFS and OS by grade of CIP. The median PFS for patients with Grade 1 was 16.4 months (95% CI: 3.5–not reached), Grade 2 was 21.2 months (95% CI: 4.2–not reached), Grade 3 was not reached (95% CI: 2.0–not reached), and Grade 5 was 1.2 months. The median OS for patients with Grade 1 was 26.6 months (95% CI: 13.0–not reached), Grade 2 was 27.4 months (95% CI: 15.6–not reached), Grade 3 was not reached (95% CI: 6. 3–not reached), and Grade 5 was 1.8 months.

The severity of CIP in response to ICI therapy.

All patients with CIP are shown in Table 4. Of the 28 patients who had CIP, 7 developed Grade 3 or higher CIP, with good treatment responses (partial response or stable disease). All these patients with severe grade CIP were treated with steroid therapy for CIP, as indicated by the American Society of Clinical Oncology clinical practice guidelines.^18^ The cryptogenic organizing pneumonia (COP) pattern was the most frequent in 19 of 28 cases (68%), including the COP +ground‐glass opacity (GGO) pattern.

**TABLE 4:** All patients with checkpoint inhibitor pneumonitis

Seq	TPS	ECOG PS^a^	Past regimens	Tumor response	CIP, Grade	Time to onset, days	Time to PD or cutoff from onset, days	Radiological pattern	Treatment	Outcome
1	NA	1	4	PR	2	122	1118	COP	Prednisolone 1 mg/kg	Improved
2	5	0	1	PR	3	141	712	COP	None	Improved
3	100	0	1	PR	2	202	651	COP	Prednisolone 0.5 mg/kg	Improved
4	100	1	0	PR	3	149	475	NOS	None	Improved
5	≧75	0	0	PR	2	21	341	COP	Steroid pulse	Improved
6	100	0	0	PR	3	150	329	NOS	Prednisolone 1 mg/kg	Improved
7	NA	1	2	PR	2	561	314	COP+GGO	Prednisolone 0.5 mg/kg	Improved
8	10	1	1	SD	2	196	279	COP	Steroid pulse	worse
9	1–24	0	1	PR	1	42	250	COP	Prednisolone 1 mg/kg	Improved
10	≧75	1	0	PR	2	21	242	COP	Prednisolone 2 mg/kg	Improved
11	70–80	0	0	PR	1	284	214	COP	Prednisolone 0.5 mg/kg	Improved
12	NA	0	1	SD	1	126	212	COP	None	Improved
13	≧75	0	1	PR	2	175	193	COP	Prednisolone 1 mg/kg	Improved
14	1–24	0	1	PR	3	107	159	COP	Prednisolone 1 mg/kg	Improved
15	0	0	3	SD	2	490	155	COP	Prednisolone 1 mg/kg	Improved
16	50–74	0	0	PR	3	141	151	GGO	Prednisolone 1 mg/kg	Improved
17	0	1	4	PR	2	224	142	COP	Prednisolone 1 mg/kg	Improved
18	60	0	0	SD	2	6	123	COP	Steroid pulse	Improved
19	1–24	1	2	SD	2	35	72	interstitial	Prednisolone 0.5 mg/kg	Improved
20	NA	0	1	PR	1	56	51	GGO	Prednisolone 1 mg/kg	Improved
21	≧75	2	0	PD	2	16	12	GGO	Prednisolone 0.5 mg/kg	Improved
22	100	0	0	PR	3	60	3	interstitial	Prednisolone 0.5 mg/kg	Improved
23	100	0	0	PR	2	857	1	COP	Prednisolone 0.5 mg/kg	Improved
24	20	1	1	SD	2	166	0	COP	Steroid pulse	Improved
25	1–24	0	1	SD	5	39	0	GGO	Prednisolone 1 mg/kg	Death
26	NA	0	3	SD	1	791	0	COP	Steroid pulse	No change
27	NA	0	1	PR	2	581	0	COP+GGO	None	Improved
28	NA	1	1	PR	2	93	0	interstitial	Prednisolone 0.5 mg/kg	No change

Abbreviations: CIP, checkpoint inhibitor pneumonitis; COP, cryptogenic organizing pneumonia‐like; ECOG PS, Eastern Cooperative Oncology Group performance status; GGO, Ground‐glass opacities; NA, not assessment, NOS, Pneumonitis not otherwise specified; PD, progression disease; PR, partial response; SD, stable disease; TPS, Tumor proportion score.

^a^ Scores range from 0 to 4, with high numbers indicating high disability.

Regarding the outcomes of CIP, there were 24 cases of improvement, 2 cases of no change, 1 case that worsened, and 1 case of death. Patient No. 25 died due to CIP; his chest imaging showed worse findings, despite the administration of steroids. The mortality rate of all the CIP grades was 3.5%.

**FIGURE 3:**
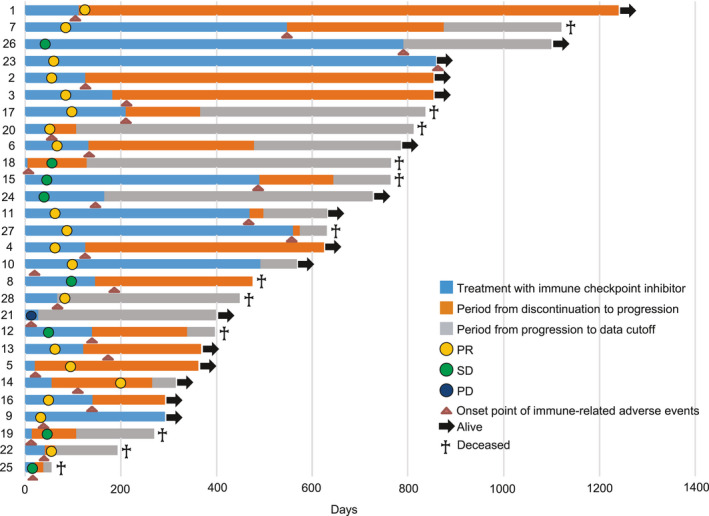
Clinical course of patients who discontinued therapy due to checkpoint inhibitor pneumonitis

A swimmer plot displaying the course of treatment for all CIP patients is shown in Figure 3. Most patients discontinued ICI treatment when they developed CIP. Treatment of CIP was conducted in 24 of 28 (86%) patients: five patients received intravenous steroid pulse therapy, and 19 patients received prednisolone 0.5–2 mg/kg therapy. CIP was improved or resolved in 24 patients. Focusing on the period from ICI treatment discontinuation to disease progression or death, seven patients lived for more than 300 days, and these patients did not show any long‐term tumor growth after treatment discontinuation (Figure 3 and Table 4; patient numbers 1, 2, 3, 4, 5, 6, and 7).

## 4 DISCUSSION

### 4.1 Overall result

In this study, we examined the correlation of CIP onset with tumor response and survival. A multivariate analysis revealed that CIP was associated with ORR, PFS, and OS. PFS and OS were longer in patients with CIP than in those without CIP, even though they had similar TTFs. To the best of our knowledge, this is the first report showing the development of CIP as an independent predictor of tumor response and survival in patients treated with anti‐PD‐1.

### 4.2 IrAEs and tumor response

Some studies have reported that irAEs are related with better outcomes in melanomas.^19^, ^20^, ^21^, ^22^ There are similar reports on lung cancer. We reported that the development of any irAE is associated with a longer PFS in patients with advanced NSCLC treated with anti‐PD‐1,^10^ and similar results were shown in other studies.^11^, ^12^, ^23^ For each irAE, we also reported that the development of skin reactions was correlated with a better response to anti‐PD‐1 antibodies.^24^ In addition, a past study reported that patients treated with pembrolizumab who developed immune‐related thyroid dysfunction had a significantly longer OS than those who did not.^25^ These findings have led to reports linking the development of any irAE or a specific irAE with clinical benefits; however, it is unknown which irAE is particularly associated with clinical benefit. In this study, we found that CIP was a significant independent predictor of clinical benefit among the irAEs by multivariate regression.

### 4.3 Checkpoint inhibitor pneumonitis

CIP is a significant adverse event that may lead to discontinuation of treatment and mortality. Symptoms of CIP are variable and nonspecific; the most common symptoms are dyspnea and cough, while fever and chest pain are less common. Furthermore, one third of patients may be asymptomatic at the onset.^14^ Therefore, members of the patient's care team need to have a high level of awareness, so that changes in the early stages of the disease are not missed. Overall, the incidence of CIP is estimated to be between 3% and 6%.^14^ However, some reports showed a higher incidence between 13% and 19%, and our results are consistent with these reports.^23^, ^26^, ^27^, ^28^ This difference in incidence may be due, in part, to the greater frequency of computed tomography (CT) in our routine clinical practice, which may have led to earlier detection, and to the inclusion of patients at potential risk for CIP who received PD‐1 inhibitors outside of clinical trials.

Radiologic features of CIP were characterized into five subtypes: COP, GGO, interstitial lung disease, hypersensitivity, and pneumonitis not otherwise specified.^14^ Viral pneumonia such as coronavirus disease, alveolar hemorrhage, and interstitial pneumonia also show non‐specific CT patterns. We need to be careful in distinguishing these diseases from CIP.^29^ In the group with CIP, TTF was shortened, but PFS and OS were prolonged. However, in a previous report, OS was significantly shortened in patients with CIP.^30^ This difference may be in the PS variation. In our study, about 4% of all patients had PS 2–4, whereas they constituted 15.2% (26/170) of the sample size in a study by Fukihara et al. This difference in findings may be due to differences in patient characteristics.

Treatment of CIP is considered to discontinue the suspect drug for pneumonitis. Patients with drug‐related pneumonitis also need to be treated with immunosuppressive drugs, but the response to systemic steroid varies with anticancer therapy.^31^, ^32^ Some cases may be fatal despite receiving appropriate therapy (Table 4). In our study, the CIP mortality rate was 3.5% (n = 1). Previous studies have also revealed a CIP‐related mortality rate ranging from 0% to 20%.^7^, ^26^, ^33^ The CIP‐related mortality in patients treated with ICIs may be lower than that in patients treated with molecular targeted therapy or conventional chemotherapy.^31^, ^32^ The different causes of pneumonitis, whether from ICI and other drugs, may have caused this difference. Pneumonitis after anticancer therapy may be caused directly by direct pulmonary toxicity or indirectly by activation of the body's inflammatory response.^34^ Most conventional chemotherapy and molecular‐targeted drugs directly injure airway epithelial cells, alveolar epithelial cells, and capillaries, resulting in non‐reversible fibrosis. However, ICI treatment may lead to the over‐activation of T cells that cause a reversible immune response in the lungs.^35^ These observations suggest that anti‐PD‐1 antibody‐induced pneumonitis might be manageable with immunosuppressive therapy.

Figure 3 shows a swimmer plot of patients with CIP. Some patients with pneumonitis showed long‐term recurrence after treatment discontinuation. A subset of patients who responded to anti‐PD‐1 in previous studies reported long‐term clinical benefit even after discontinuation of therapy.^36^ In this study, although OS in patients with CIP was longer than in those without CIP, the factors are not clear. This good outcome may be related to early diagnosis and treatment, which ensures proper management at a less severe stage. We have an irAE management team (frontline immunotherapy team [FIT]) that enables us to perform a thorough physical examination and to report and treat even mild symptoms as early as possible.

Presently, the mechanism by which the antitumor effect persists even after interruption of treatment with ICI has not been clarified. Osa et al. assessed the time of maximum duration of antibodies on T cells and the relationship between this duration and residual therapeutic effect or potential adverse events. They reported that the binding of nivolumab to memory T cells in the blood was detectable more than 20 weeks after the last dose, irrespective of the number of nivolumab doses or subsequent treatments.^37^ This mechanism suggests that long‐term antitumor effects may be sustained. Therefore, appropriate management of CIP may have a good and long therapeutic effect.

### 4.4 Limitations

There are a few limitations to our study. The study had a small sample size, was a non‐randomized, single‐center cohort, retrospective study. Also, the expression level of PD‐L1 was not measured because of the commercial unavailability of diagnostic kits at the beginning of the study in Japan. Hence, we were not able to sufficiently evaluate the therapeutic effect of PD‐L1 expression. Recently, the combination of chemotherapy and immunotherapy has become the norm, reducing the chances of treating patients on anti‐PD‐L1 monotherapy. However, the results of this study may be useful in predicting the clinical efficacy of chemotherapy–immunotherapy combination therapy or immunotherapy combination therapy.

## 5 CONCLUSION

In our study, CIP was associated with prolonged PFS and OS. Moreover, in 25% of patients with CIP, tumors did not grow long after treatment was discontinued. Most importantly, management was carried out at the time of CIP onset and we believe that careful CIP management, especially early detection and treatment, will ease the attainment of the maximum clinical efficacy from anti‐PD‐1 antibody monotherapy.

## CONFLICT OF INTEREST

Dr. Sugawara reports lecture fees from Ono Pharmaceutical, Bristol‐Myers Squibb, MSD, AstraZeneca, Chugai Pharma, Nippon Boehringer Ingelheim, Pfizer, Taiho Pharmaceutical, Eli Lilly and Company, Novartis, and Kyowa Hakko Kirin. Dr. Toi reports lecture fees from Ono Pharmaceutical, Bristol‐Myers Squibb, MSD. Dr. Saito reports lecture fees from Bristol‐Myers Squibb. Dr. Domeki reports lecture fees from Ono Pharmaceutical, Bristol‐Myers Squibb. Dr. Nakamura reports lecture fees from MSD.

## AUTHOR CONTRIBUTIONS

Drs Ono K, Toi, and Sugawara had full access to all of the data in the study and take responsibility for the integrity of the data and the accuracy of the data analysis. *Study concept and design*: Ono K, Toi, and Sugawara. *Acquisition, analysis*, *or interpretation of data*: Ono K, Ono H, Toi, Sugawara, Sugisaka, Kawashima, Aiba, Kawana, Saito R, Aso, Odaka, Matsuda, Saito S, Narumi, Tsurumi, Shimizu, Domeki, Terayama, Nakamura, Yamanda, Kimura, and Honda. *Drafting of the manuscript*: Ono K, Toi, and Sugawara. *Critical revision of the manuscript for important intellectual content*: Ono K, Ono H, Toi, Sugawara, Sugisaka, Kawashima, Aiba, Kawana, Saito R, Aso, Odaka, Matsuda, Saito S, Narumi, Tsurumi, Shimizu, Domeki, Terayama, Nakamura, Yamanda, Kimura, and Honda. *Statistical analysis*: Ono K, Toi, and Sugawara.

## ETHICAL APPROVAL FROM AN INSTITUTIONAL REVIEW BOARD

The study was conducted with the approval of the Institutional Review Board of Sendai Kousei Hospital (IRB No. 30–36).

## Data Availability

All data generated or analyzed during this study are included in this published article.
